# 3D Printing of ABS Barium Ferrite Composites

**DOI:** 10.3390/ma13061481

**Published:** 2020-03-24

**Authors:** Thomas Hanemann, Diana Syperek, Dorit Nötzel

**Affiliations:** 1 Institute for Applied Materials, Karlsruhe Institute of Technology, D-76344 Eggenstein-Leopoldshafen, Germany; dorit.noetzel@kit.edu; 2 Department of Microsystems Engineering, University Freiburg, D-79110 Freiburg, Germany; diana.syperek@googlemail.com

**Keywords:** 3D printing, fused filament fabrication (FFF), magnetic composites, additive manufacturing of polymer matrix-ceramic-composites

## Abstract

In this work, a process for the realization of new polymer matrix composites with nanosized barium ferrite (BaFe_12_O_19_) as ferrimagnetic filler, acryl butadiene styrene (ABS) as polymer matrix and an extrusion-based method, namely fused filament fabrication (FFF), as 3D printing method will be described comprehensively. The whole process consists of the individual steps material compounding, rheological testing, filament extrusion, 3D-printing via FFF and finally a widespread specimen characterization regarding to appearance, mechanical properties like tensile and bending behavior as well as the aspired magnetic properties. Increasing ferrite amounts up to 40 vol.% (equal 76 wt.%) cause a reduction of the ultimate stress and an increase of the magnetic polarization as well as of the energy product (BH)_max_ in comparison to the pure polymer matrix. In addition, an extensive discussion of typical printing defects and their consequences on the device properties will be undertaken.

## 1. Introduction

The different variants of additive manufacturing, or more popular named 3D printing, allows, nowadays, the realization of parts with geometrical features, which cannot be produced by applying classical or established fabrication methods. Related to processing, the additive manufacturing methods can be divided into seven categories, namely material extrusion, material jetting, binder jetting, powder bed fusion, direct energy deposition, vat polymerization and sheet lamination [1]. Starting approximately 30 years ago with stereolithography (SLA), a huge number of different methods have been invented and developed. Amongst others, material extrusion-based methods are very popular, especially fused filament fabrication (FFF), same is valid for 3D inkjet printing (also known as PolyJet or Multijet), or the variants of powder based printing (binder jetting, laser sintering, et al.). From a materials point of view the 3D printing of polymerizable resins, like in SLA, or extrudable polymer melts, is widely distributed and has a recognized technological readiness level [2,3,4,5]. In case of the extrusion-based techniques, especially FFF, recently the portfolio of applicable thermoplastics has been expanded from low and medium performance materials like polylactide (PLA) or acryl butadiene styrene (ABS) upwards to high performance polymers, like polyetheretherketone (PEEK) [6]. This is attributed to a significant progress in the accessible printer’s process parameters like maximum printing temperature up to 500 °C and more. Current research focuses on the realization of multi-material printing systems and application in life science, biology and medicine [7,8,9] applying FFF and other 3D printing technologies. For the dissemination of 3D printing into new fields, the adaption of the printing process to non-standard materials is mandatory, like in construction [10] or the exploitation of mechanical properties in devices applying metals or ceramics [11,12,13]. In addition to the main material classes and beyond thermomechanical properties, there was a strong development in FFF towards new polymer matrix composites with additional functionalities, like conductive, dielectric, ferroelectric, magnetic and a.o. properties [14,15,16,17]. The FFF printing process, especially the printing parameters and the printing strategy, have a pronounced impact not only on the resulting mechanical but also on the aspired functional properties and appearance [18,19,20].

The aim of this work is the presentation and critical discussion of the FFF 3D printing process development applying a new ABS-nanosized barium ferrite-composite for the realization of magnetic polymer matrix materials. Furthermore, an extensive discussion of typical printing defects and their consequences on the device properties will be undertaken. Preliminary results dealing with the process chain development without further detailed data analysis of this work have been presented earlier during the Microsystem Technology Congress 2019 in Berlin [21].

## 2. Materials and Methods

For the fabrication of via FFF printed magnetic specimen a process chain consisting of the individual steps has been utilized.

1. Material selection
2. Compounding and rheological characterization
3. Filament extrusion
4. Composite printing
5. Specimen characterization

At each step, a comprehensive material or specimen characterization was performed with respect to find a robust process description. As in previous work dealing with the realization of FFF printed specimen, the following sample preparation strategy was pursued [16,17]: The ceramic yield was increased up to a value with a pronounced torque or viscosity gain relative to the pure polymer matrix. Using ABS as host polymer matrix solid volume loads around 35–50 vol.% could be achieved by compounding [16,17]. In the past FFF composite printing was the limiting process step, depending on the particle characteristics specimen with a solid load only around 30–40 vol.%, which were printable in an acceptable quality suitable for mechanical or functional property testing [16,17].

### 2.1. Material Selection and Characterization

Commercial ABS was selected as thermoplastic matrix material, to ensure good FFF printing behavior, an easy flowing injection molding grade was selected (Terluran GP-35, INEOS Styrolution, Frankfurt, Germany). Nanosized barium ferrite (BaFe_12_O_19_, Nanostructured and Amorphous Materials Inc., Katy, TX, USA) as magnetic material. Stearic acid (Sigma-Aldrich, Munich, Germany) with a concentration of 3.3 mg/m^2^ filler’s specific area was chosen as surfactant for better composite homogenization and viscosity adjustment. The vendor of the used barium ferrite quote an average particle size around 500 nm. The particle size distribution was measured in isopropanol using a Microtrac X100 (Microtrac MRB, Haan, Germany), the specific surface area with a Flow Sorb II 2300 (Micromeritics Instruments, Norcross, GA, USA). All scanning electron microscopy (SEM) images were recorded with Carl Zeiss Supra 55 (Carl Zeiss Microscopy GmbH, Jena, Germany).

### 2.2. Compounding and Rheological Characterization

A mixer-kneader unit (W50-EHT, Brabender, Duisburg, Germany) with torque recording was used for composite formation. The mixing temperature was set to 210 °C, the mixing time to 60 min. For better comparison, a fixed sequence of the individual components addition into the mixer-kneader was defined: First, approximately 20% of the filler was placed in the mixing chamber, followed by the surfactant for improved surface coverage. Then the whole amount of ABS was added, finally the remaining ceramic was filled in. The rheological properties were investigated by applying a Rheograph 25 (Goettferd, Buchen, Germany) at a measuring temperature of 240 °C and shear rates up to 6000 1/s. The effective solid load of all composites along the whole process chain were controlled by thermographimetric analysis (Netzsch STA406, NETZSCH Group, Selb, Germany) with heating rates of 10 K/min under air atmosphere.

### 2.3. Filament Extrusion

Prior to filament extrusion all composites were dried at 80 °C (universal oven, Memmert, Schwabach, Germany) for at least 2 h. A Noztek Pro High Temperature Extruder (Shoreham, West Sussex, UK) with a cylinder and nozzle temperature of 210 °C was used for filament extrusion. All filaments showed after extrusion a diameter of 1.75 ± 0.1 mm.

### 2.4. Composite Printing

All specimens were printed on a Makerbot 2X FFF (Makerbot Ind. Brooklyn, NY, USA) printer. The printing platform temperature was set to 110 °C, the nozzle diameter was 0.4 mm and the printing speed was varied between 30 and 90 mm/s to obtain samples with as less as possible defects. The extrusion temperature was set to 230 °C, for solids loads larger than 20 vol.% an extrusion temperature of 240 °C was necessary. The temperature of the built platform was fixed to 110 °C for good specimen adhesion during printing. Due to software restrictions, only a 90° orientation of the printing direction relative to the previous one was possible. With respect to characterization five up to ten samples (mechanical testing) and five (magnetic testing) were printed for each composition.

### 2.5. Specimen Characterization

The optical appearance of the filaments and the specimen were controlled with stereo microscopes SZ61 and BX61, both from Olympus (Hamburg, Germany). All polished longitudinal cuts and cross-sections were done using a Buehler (Esslingen, Germany) Phoenix 4000 polishing machine with the aid of SiC 180 sand paper (2–5 min, 150 rpm and 5 bar grinding pressure) and subsequent polishing with 1 µm diamond suspension (5 min, 150 rpm and 5 bar grinding pressure). All test geometries and selected test parameters are shown in Table 1. The tensile tests were performed according to ASTM D 638 Type IV applying a Zwick/Roell (Ulm, Germany) universal testing machine Z010. The bending tests followed ASTM D790 in a three-point setup using a Zwick Z005 testing machine. The magnetic properties were investigated at a service company using a Permagraph C-500 Fluxmeter (Steingroever, Koeln, Germany) following DIN IEC 60404-5; due to cost reasons, only one specimen per composition was measured; hence, a statistical evaluation is not available and the obtained values should be treated as orientation values. The experimental error of the used equipment is around 1%.

## 3. Results and Discussion

### 3.1. Material Properties and Characterization

**Figure 1:**
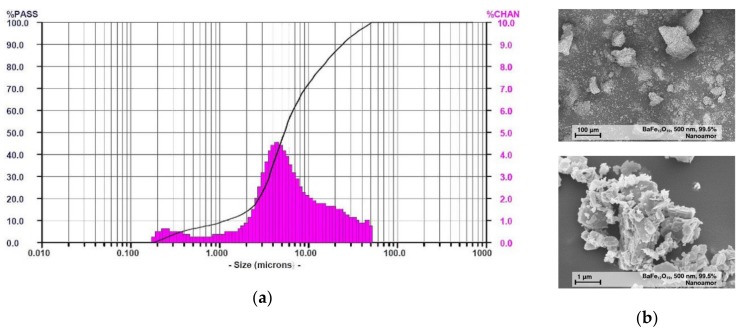
(**a**) measured particle size distribution; (**b**) SEM images at different magnification of barium ferrite crystallites. The particles are highly agglomerated and show an irregular shape [22].

The particle properties (particle size distribution, specific surface area (BET)) have been reported earlier [22]; the used barium ferrite shows a d_10_-value of 1.2 µm, a d_50_-value of 5.4 µm and a d_90_-value of 24.1 µm (Figure 1a). The BET was measured to be 12.2 m^2^/g. SEM-images gave a strong evidence for a pronounced particle agglomeration and non-spherical shapes, which may cause difficulties during compounding (Figure 1b).

### 3.2. Compounding and Rheological Characterization

**Figure 2:**
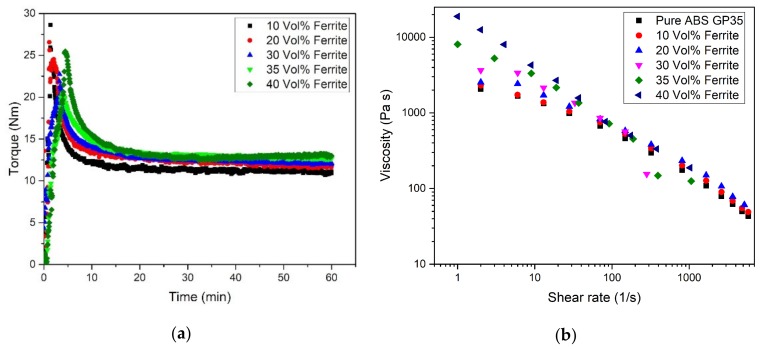
(**a**) resulting torque during compounding as function of compounding time and solid load; (**b**) shear rate and solid load dependent viscosity (at 240 °C).

Compounds consisting of ABS as polymer matrix, stearic acid as surfactant and increasing amounts of barium ferrite were prepared by a mixer-kneader with torque recording. Following earlier investigations [16,17], a huge solid load (denoted as vol.%) is targeted to achieve significant property changes. Due to the large ferrite density (5.4 g/cm^3^) a high ceramic weight content is necessary to obtain a huge volume content, e.g., 35 vol.% is equivalent to 72 wt.%. As known from previous investigated composites with e.g., ABS as polymeric host, the torque increases with increasing solid load [16,17] (Figure 2a). The measured compounding curve can be split into three different time regimes, roughly [23]: In the first 5–7 min (filling state) all components (ABS, ceramic and dispersant) are added, as described in the experimental section. The filling state is characterized by a huge initial torque values due to direct particle-particle interaction. The first regime ends with the complete addition of all components and delivers non-specific maximum torque values. In the second regime (mixing state), the particles deagglomerates and are wetted by the surfactant, delivering a pronounced torque drop. Finally, a steady state (equilibrium state) is reached with a complete wetting of the ceramic particles by the surfactant and the polymer. Increasing solid loadings extend the filling time, which can be depicted from the compounding time until the maximum torque value is reached. With respect to composite homogeneity, the numerical value of the maximum torque is without any particular meaning for the final composite homogeneity as described earlier [23]. An increase of the solid load causes an increasing particle–particle interaction delivering higher equilibrium torque values according to enhanced inner friction. Comparable results can be derived from the rheological investigations (Figure 2b). Pure ABS and all composites show a pronounced pseudoplastic flow, which means a significant viscosity drop with applied shear rate at constant temperature. Especially at shear rates below 100 1/s increasing ferrite load causes a pronounced viscosity increase shifting the zero shear viscosity to smaller shear rates and higher viscosities [24]. This pseudoplastic flow behavior is favorable in injection molding due the large injection pressure and injection speed, but adverse for the more or less pressureless FFF. Typical feedstocks used in ceramic injection molding with a solid load around 50 vol.% show at injection temperature and realistic shear rates larger than 1000 1/s viscosity values smaller than 100 Pa·s [25]. In case of FFF typical shear rates are in the range of 50–200 1/s depending on the used extrusion nozzle and printing speed at the given printing temperature [26]; hence viscosity values around 500–1000 Pa·s are realistic even for composites with low filler load. From the measured viscosities values it can be expected, that with increasing ferrite content FFF printing could be difficult.

### 3.3. Filament Extrusion

**Figure 3:**
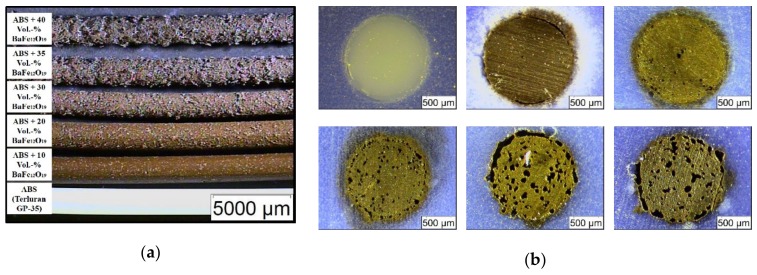
Microscopic images: (**a**) extruded filaments with different solid loadings; (**b**) polished cross-sections with increasing filler content: 1. Row: Pure acryl butadiene styrene (ABS), 10 vol.%, 20 vol.% ferrite, 2. Row: 30 vol.%, 35 vol.% and 40 vol.% ferrite.

A commercial filament extruder was taken for filament fabrication, due to pronounced extrudate swelling of the pure ABS, a 1.4 mm extrusion nozzle was applied; in case of all composites, the 1.8 mm extrusion nozzle was used. Surprisingly, starting at a filler load of 20 vol.% a pronounced poor filament surface quality could be observed, which deteriorated significantly with increasing ceramic content (Figure 3a). Even at a low ceramic load of 10 vol.% an inhomogeneous surface can be seen; at higher loads a badly jointed appearance is obvious. This may be attributed to the addition of the ceramic filler, which may be justified with the non-spherical morphology and the broad particle size distribution due to agglomeration presented in Figure 1.

**Figure 4:**
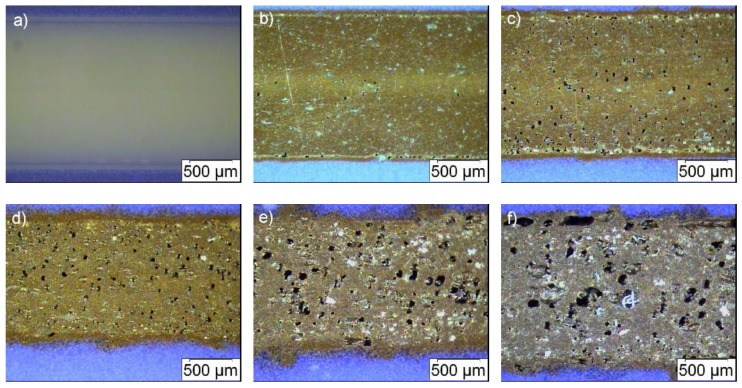
Microscopic images of polished longitudinal cuts with increasing filler content: Upper row, from left to right: Pure ABS (**a**), 10 vol.% (**b**) and 20 vol.% (**c**); lower row: 30 vol.% (**d**), 35 vol.% (**e**) and 40 vol.% (**f**) ferrite.

Polished cross-sections of all filaments (Figure 3b) showed at higher loads, in addition to the observed surface inhomogeneities, an increasing occurrence of voids inside the filament, and in accordance to the surface appearance, the presence of an outer shell around the filament core. This result is quite surprising, because it was never observed before after compounding using ABS or other highly filled polymer matrix systems for powder injection molding or FFF like wax/polyethylene or polyethyleneglycol/polyvinylbutyral mixtures. Hence, it should be attributed to the applied filler, may be in the second step (mixing state) of compounding, the shear forces during compounding were not sufficient to deagglomerate the particles. Consequently, only agglomerates, which contain voids between the individual primary particles, are coated with the surfactant first and second with the polymer; unfortunately, this behavior cannot be depicted from the torque vs. time presentation (Figure 2a). The agglomerate coating instead of primary particle coating may explain the presence of an increasing number of voids in the filament with increasing solid load and the inhomogeneous filament appearance. For a better evaluation of the filament quality, polished longitudinal cuts have been prepared (Figure 4). Unfortunately, the poor filament constitution has been confirmed showing a huge volumetric amount of voids and a broken up outer shell.

### 3.4. Composite Printing

**Figure 5:**
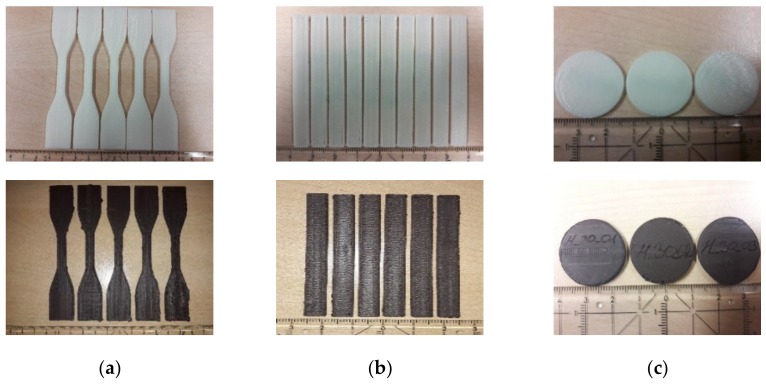
Printed test specimen, upper row pure ABS, lower row composite with 30 vol.% ferrite: (**a**) Tensile test; (**b**) Bending test; (**c**) Magnetic measurement.

**Figure 6:**
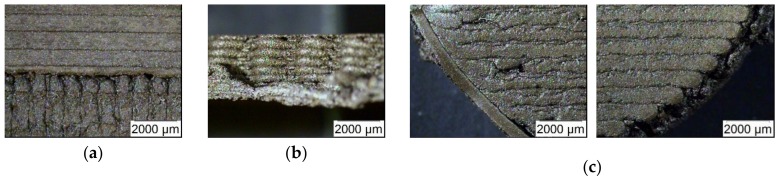
Printing defects observed at a printed composite with 30 vol.% ferrite content: (**a**) top view tensile test specimen, contact between clamping and gauge zone; (**b**) side view tensile test specimen, missing envelope; (**c**) top view magnetic specimen with voids and incompletely filled envelope.

All composite filaments have been tested for FFF printing applying the Makerbot 2X printer. Due to the large viscosity, the composite with 40 vol.% could not be extruded in a proper way and therefore no specimen could be printed. In all other cases the shells of the specimen were printed first, the infill was set to 100%. For a certain quality control between 5 and 10 test samples were produced for the mechanical characterization, in case of the magnetic measurements 5 specimen were sufficient. Pure ABS samples were fabricated as reference. Specimen in sufficient quality with 35 vol.% solid load could only be printed for bending and magnetic test purposes. Figure 5 shows printed specimen, in each case pure ABS as reference and a composite with 30 vol.% solid load. A closer look to the printed specimen showed especially for the composites with higher ceramic content significant printing defects (Figure 6), which can be attributed to the huge composite viscosity, the poor filament quality, the pressureless deposition and a not optimized printing strategy in combination with the printer’s accuracy specification.

In all cases, voids within the structure and at the transition from infill to envelope can be seen as well as the layer-by-layer filament deposition within one layer and between subsequent deposited layers. The observed voids are typical for by FFF printed parts and can be found in current literature as well investigating composites and sintered metals [27,28,29,30], also observed in case of magnetic filler dispersed in a polymer matrix [31]. The presence of voids in the printed samples influences all physical properties: In case of mechanical testing, the mechanical properties are reduced because the voids acts as defect volume. In case of e.g., dielectric properties, the relative permittivity value of air (ε_rel_ ≈ 1) lowers the specimen sum permittivity value obtained by the barium titanate and the polymer according to their volume fraction and individual relative permittivity values [16].

### 3.5. Specimen Characterization

#### 3.5.1. Mechanical Characterization

As expected, the addition of the filler has a pronounced impact on the mechanical properties. In contrast to e.g., injection molded test specimen with defect free samples the measured values cannot be attributed only to the composite composition. Due to previous discussed specific FFF process characteristics of layer-by-layer deposition and the round shape of the filaments, defects like voids and lamellar structures with a reduced adhesion between the layers can be expected, which deteriorates the mechanical properties significantly.

**Figure 7:**
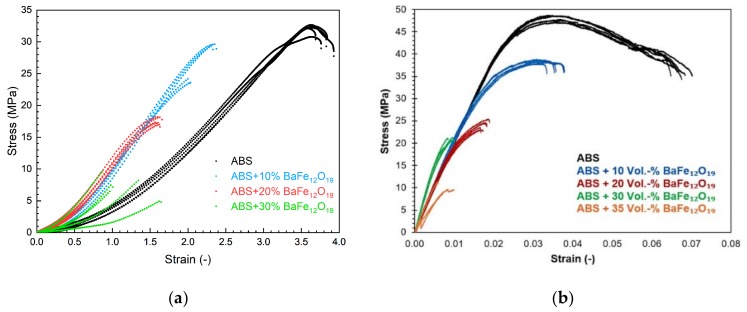
Stress–strain behavior (**a**) tensile test; (**b**) bending test.

**Figure 8:**
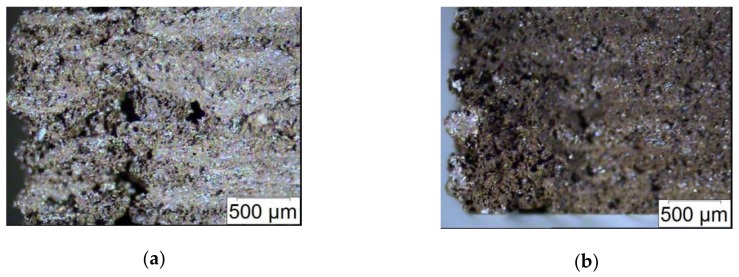
Specimen cross section after mechanical testing of a composite with 30 vol.% ferrite: (**a**) tensile test and (**b**) bending test.

Figure 7 shows the results derived from tensile (a) and bending tests (b), furthermore Table 2 lists the accessible ultimate stress as function of the ferrite load in the composite. Increasing solid loadings have a certain impact on the mechanical properties: First the tensile strength is reduced, also the ultimate stress and ultimate strain. Second and especially obvious in case of the composite with 30 vol.% ferrite, the measurements show some scattering. The measured stress–strain behavior is a superposition of two effects: On the one hand the particles act as discontinuities in the polymer matrix, on the other hand the printing process itself generates defects in the specimen. Both mechanisms delivers a non-homogeneous bulk material with some property scattering. A closer view on the bulk material show, that both typical type of defects—voids within one layer as well as between layers and delamination of printed layers—can be found in the specimen (Figure 8). This is in agreement with results also published for FFF-fabricated magnetic composites [31,32]. The highly filled specimens are very fragile to mechanical stress, so in case for future applications as magnetic composite a stress-free environment must be selected.

#### 3.5.2. Magnetic Characterization

**Figure 9:**
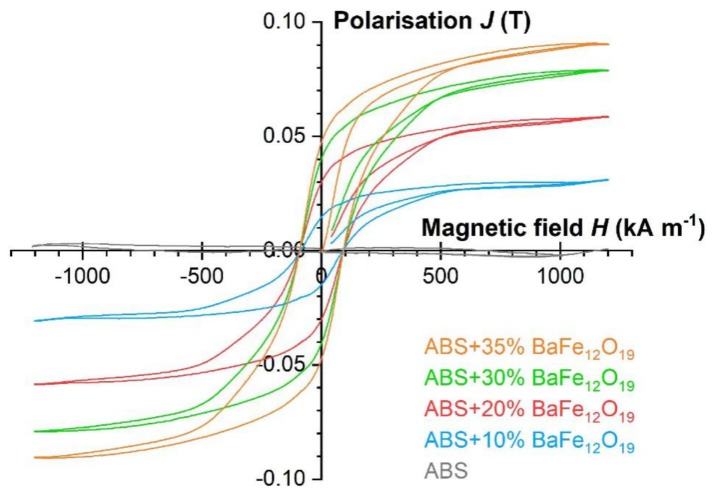
Change of the magnetic polarization as function of the applied magnetic field and ferrite content.

All measured magnetic properties as function of the barium ferrite are summarized in Table 3. Though only one sample was investigated per composition, a clear correlation between solid load and magnetic response is detectable, also the hysteresis under magnetization (Figure 9). The absolute values for the remnant flux density B_remn_, the polarization J_max_ as well as for the energy product (BH)_max_ are moderate even for the highest ferrite content, which can be attributed to the huge number of voids in the samples.

Recent results describes FFF of strontium ferrite, dispersed in polyamide 12 up to a solid load of 55 vol.%, printed on the surface on an alignment magnet (NdFeB), which enhances the magnetic properties due to external orientation in z-direction. Remnant magnetic flux densities around 178 mT for a solid load of 40 vol.% and 213 mT for 55 vol.% could be achieved [33]. Without external magnetic field orientation values around 102 mT (40 vol.%) and 149 mT (55 vol.%) were measured [33]. Wei et al. investigated the magnetic properties of barium and strontium ferrite, dispersed in a polyvinylalcohol/polyethylene-glycol (PVA/PEG) binder, applying a syringe-type 3D printing process and different ferrite treatment strategies [34]. Palmero et al. investigated the filament fabrication and magnetization of composites consisting of small amounts of strontium ferrite (8 wt.%) and NdFeB (6.6 wt.%), dispersed in ethylene ethyl acetate [35]. Composites consisting of 65 vol.% coarse grained (20–200 µm) NdFeB plate-shaped particles, dispersed in polyamide 12, delivered huge values for the remnant flux density of 0.51 T and an energy product of 43.49 kJ/cm^3^ applying material extrusion [36]. Nagarajan et al. investigated the impact of an applied magnetic field during SLA of a photocurable dispersion containing 1 wt.% strontium ferrite [37]. Powder bed fusion can also be used for the additive manufacturing of magnets using a NiMnGa alloy [38]. In contrast to the above listed values described in literature, the obtained experimental results are moderate, if one compares for example the measured remnant flux density of 47 mT (at 35 vol.%) with the 102 mT (at 40 vol.% strontium ferrite in PA12), described in reference [33], which can be attributed to the poor sample quality.

#### 3.5.3. Process Characterization

**Figure 10:**
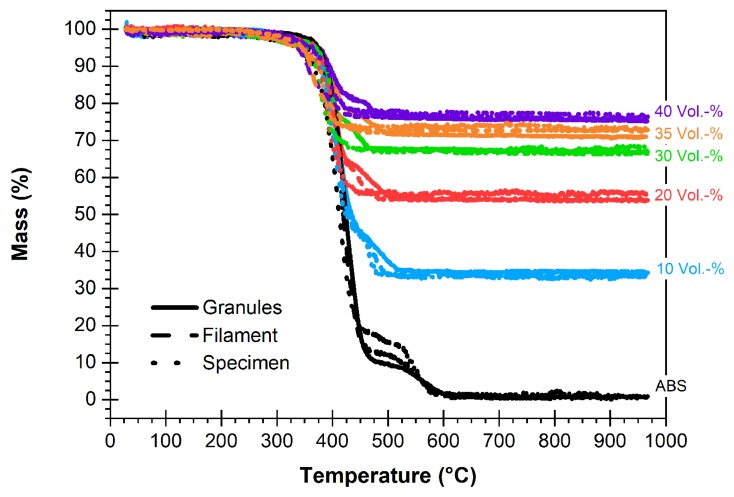
Validation of the consistency of the composite composition during processing via thermographimetric analysis.

With respect to the development of a robust process, it has to be proved that the different individual manufacturing steps do not alter the initial balanced composite’s solid load. The ferrite content was measured by thermal analysis after pelletizing, filament formation and finally FFF printing. Figure 10 shows the temperature dependent weight loss of all investigated composites and the polymer matrix ABS as reference. In all cases, the main polymer decomposition occurs in the temperature range between 350 and 450 °C. Within the experimental error no relevant deviation of the solid load during processing can be detected, increasing ferrite amounts may cause a slightly reduced polymer decomposition temperature. In the temperature range between 450–550 °C, an influence of the processing can be observed even for the pure polymer matrix; one has to consider, that during manufacturing each processing step means a high additional thermal stress (compounding: 210 °C, rheological measurement: 240 °C, filament fabrication: 210 °C and printing: 220–240 °C), which may accelerate thermal decomposition.

## 4. Conclusions and Outlook

Within the frame of this work it was shown, that a barium ferrite ABS composites can be realized by compounding, filament formation and 3D Printing via FFF. It was possible to prepare composites up to a ferrite load of 40 vol.% (equal 76 wt.%) and to characterize the rheological properties as function of shear rate and solid load. Unfortunately, the used low-cost FFF printer was not able to print the 40 vol.% composite due to technical deficiencies. The mechanical characterization of the specimen yielded a pronounced deterioration of the mechanical properties with solid load. This can be attributed to two different sources, namely the particle properties and the printing process. First, the barium ferrite particle properties, especially the morphology and the state of agglomeration, are disadvantageous for composite formation due the irregular crystallite appearance. Second, the FFF printing process delivers discontinuities within one printed layer and between printed layers in the specimen. With respect to the aspired magnetic properties a clear correlation between ferrite content and magnetic properties could be observed, again the voids in the specimen reduces the sum magnetization significantly. For further property improvement different alterations should be undertaken in future. First, a magnetic material with larger particle sizes and spherical shape should be used avoiding the observed enhanced void formation and poor filament homogeneity and second a polymer matrix with lower melt viscosity reducing the defect formation during FFF printing. Third, the assignment of an enhanced FFF printer with better temperature control during printing and improved accuracy in x,y,z-directions should minimize the voids coming from the printing process. Finally, and depending on the aspired devices, the use of an external magnetic alignment field close to the FFF printing nozzle or at the built platform should enhance the magnetic properties significantly.

## Figures and Tables

**Table 1:** Specimen geometries and selected test parameters.

Feature	Tensile Tests	Bending Tests	Magnetic Measurement
Applied standard	ASTM D638 Type IV	ASTM D790	DIN IC 60404-5
Specimen geometry	length × height × width 115 × 3.2 × 19 (mm^3^)	length × height × width 72 × 6.5 × 27.3 (mm^3^)	Diameter × height 25 × 2 (mm^2^)
Gauge length	25 mm	n.a.	n.a.
Gauge width	6 mm	n.a.	n.a.
Measuring speed	5 ± 1.25 mm/min	1 mm/min	n.a.
Temperature	25 ± 2 °C	25 ± 2 °C	23.5 ± 0.4 °C
Relative humidity	50 ± 5%	50 ± 5%	n.a.

**Table 2:** Ultimate stress (average values) of all composites, derived from tensile and bending tests.

Composition	Tensile Stress (MPa)	Number of Samples	Bending Stress (MPa)	Number of Samples
ABS	32.3 ± 2.0	10	35.3 ± 0.8	10
ABS/10 vol.% Ferrite	26.5 ± 3.3	6	35.8 ± 0.2	7
ABS/20 vol.% Ferrite	17.5 ± 0.6	7	23.3 ± 0.9	6
ABS/30 vol.% Ferrite	7.5 ± 1.8	5	20.3 ± 0.9	6
ABS/35 vol.% Ferrite	-	-	9.7 ± 1.2	3

**Table 3:** Magnetic properties of all investigated ABS ferrite composites.

Composition	H_max_ (kA/m)	J_max_ (mT)	B_remn_ (mT)	H_coer_ (kA/m)	(BH)_max_ (kJ/m^3^)
Without sample	1201	2	0.4	0.4	0
ABS	1201	1	0.6	0.4	0
ABS/10 vol.% Ferrite	1201	31	15	11	0.04
ABS/20 vol.% Ferrite	1202	59	30	20	15
ABS/30 vol.% Ferrite	1201	79	40	25	26
ABS/35 vol.% Ferrite	1202	91	47	29	35
